# An Electronic Patient-Reported Outcomes Tool for Older Adults With Complex Chronic Conditions: Cost-Utility Analysis

**DOI:** 10.2196/35075

**Published:** 2022-04-20

**Authors:** Rafael N Miranda, Aunima R Bhuiya, Zak Thraya, Rebecca Hancock-Howard, Brian CF Chan, Carolyn Steele Gray, Walter P Wodchis, Kednapa Thavorn

**Affiliations:** 1 Institute of Health Policy, Management and Evaluation University of Toronto Toronto, ON Canada; 2 Toronto Health Economics and Technology Assessment Collaborative University Health Network Toronto, ON Canada; 3 KITE - Toronto Rehabilitation Institute University Health Network Toronto, ON Canada; 4 Bridgepoint Collaboratory for Research and Innovation Lunenfeld-Tanenbaum Research Institute Sinai Health Toronto, ON Canada; 5 Institute for Better Health Trillium Health Partners Toronto, ON Canada; 6 Clinical Epidemiology Program Ottawa Hospital Research Institute Ottawa, ON Canada; 7 School of Epidemiology and Public Health University of Ottawa Ottawa, ON Canada

**Keywords:** eHealth, multimorbidity, primary care, cost-effectiveness, older adult, elder, cost, patient reported outcome, community, complex care, aging, Canada, North America, chronic disease, chronic condition, decision tree, model, sensitivity analysis

## Abstract

**Background:**

eHealth technologies for self-management can improve quality of life, but little is known about whether the benefits gained outweigh their costs. The electronic patient-reported outcome (ePRO) mobile app and portal system supports patients with multiple chronic conditions to collaborate with primary health care providers to set and monitor health-related goals.

**Objective:**

This study aims to estimate the cost of ePRO and the cost utility of the ePRO intervention compared with usual care provided to patients with multiple chronic conditions and complex needs living in the community, from the perspective of the publicly funded health care payer in Ontario, Canada.

**Methods:**

We developed a decision tree model to estimate the incremental cost per quality-adjusted life year (QALY) gained for the ePRO tool versus usual care over a time horizon of 15 months. Resource utilization and effectiveness of the ePRO tool were drawn from a randomized clinical trial with 6 family health teams involving 45 participants. Unit costs associated with health care utilization (adjusted to 2020 Canadian dollars) were drawn from literature and publicly available sources. A series of sensitivity analyses were conducted to assess the robustness of the findings.

**Results:**

The total cost of the ePRO tool was CAD $79,467 (~US $ 63,581; CAD $1733 [~US $1386] per person). Compared with standard care, the ePRO intervention was associated with higher costs (CAD $1710 [~US $1368]) and fewer QALYs (–0.03). The findings were consistent with the clinical evidence, suggesting no statistical difference in health-related quality of life between ePRO and usual care groups. However, the tool would be considered a cost-effective option if it could improve by at least 0.03 QALYs. The probability that the ePRO is cost-effective was 17.3% at a willingness-to-pay (WTP) threshold of CAD $50,000 (~US $40,000)/QALY.

**Conclusions:**

The ePRO tool is not a cost-effective technology at the commonly used WTP value of CAD $50,000 (~US $40,000)/QALY, but long-term and the societal impacts of ePRO were not included in this analysis. Further research is needed to better understand its impact on long-term outcomes and in real-world settings. The present findings add to the growing evidence about eHealth interventions’ capacity to respond to complex aging populations within finite-resourced health systems.

**Trial Registration:**

ClinicalTrials.gov NCT02917954; https://clinicaltrials.gov/ct2/show/NCT02917954

## Introduction

Community-dwelling older adults (≥ 65 years old) with multiple chronic conditions and complex care consume a substantial amount of health care resources [1,2]. Existing evidence has shown that patients with multimorbidity have more frequent hospital admissions, longer hospital stays, and that health care costs exponentially increase with the greater number of health conditions, placing a high economic burden on health systems [3,4]. Provision of care is particularly challenging for this population due to the lack of specific assessment tools for multimorbidity, and the more complex management and coordination of care, which involves different professionals and clinical settings. The difficult management of multimorbidity—with guidelines that focus on single conditions, multiple therapies, and medications—can reduce treatment adherence and patients’ health-related quality of life (HRQoL) [2,4,5]. Moreover, older adults are at a higher risk of poor health outcomes given the complexity of social, environmental, and other contextual issues that they face within and outside the health system, such as social frailty and access to home and community care that meet their needs [6,7].

To address these challenges, there is growing interest in person-centered, integrated, and holistic care approaches that may help coordinate personalized and comprehensive care involving older adults, their caregivers, and health care providers [4,8]. Additionally, self-management programs have created efficiency gains, yielding improvements in health status and reductions in unnecessary health care utilizations [8]. However, there are few existing digital tools to enable these person-centered approaches for older adults in primary care settings [9]. The electronic patient-reported outcome tool (henceforth, called the ePRO tool) is one of such digital tools, which can facilitate collaborative care based on individualized goals created by older adults and providers, also known as goal-oriented care. The tool is delivered through the internet and mobile devices, and can be useful for complex care given their ability to improve access, continuity and efficiency of care, patient self-management, and communication [10].

A randomized trial had shown that ePRO plus usual care did not significantly improve the HRQoL in older adults with complex needs, partly due to recruitment challenges [10]. However, ethnographic data collected as part of the trial highlighted the importance of the coherence or meaningfulness of the intervention to the end users (ie, patients and providers) and uncovered the challenge to align coherence across diverse groups. When coherence was well aligned, users were more likely to see the value of the technology and use it more over time. In addition to assessing perceived value, there is a need to examine whether the challenges to improve clinical outcomes balance the additional investment in provider and technology costs associated with administering the ePRO tool within a clinical setting. While previous studies have shown eHealth interventions to be cost-effective [11], the cost-effectiveness of the ePRO tool, which was implemented in the community setting, has not been formally evaluated. This study was therefore conducted to estimate the cost of ePRO tool and examine whether the benefits gained from the tool outweighed its costs from the perspective of Canada’s publicly funded health care system.

## Methods

### Study Design and Population

We performed a cost-utility analysis of the ePRO compared with standard care. The analysis was based on data from a pragmatic, stepped-wedge, cluster randomized trial with patients from 6 comprehensive primary care practices—called family health teams (FHTs)—across Ontario, Canada. FHTs provide integrated primary care, led by a physician or a nurse practitioner, and assisted by other professionals such as registered nurses, social workers, and dietitians [12]. A usual care pathway for older adults with multiple chronic conditions may include routine visits to their health care providers with or without their caregivers.

All FHT sites started in the control period, during which all recruited patients received usual care, and were randomly assigned to either the early or late intervention groups, with an initial control period of 3 and 6 months, respectively. The FHTs were then switched to the intervention period, during which patients and providers used ePRO as part of the primary care, for 12 months in the early intervention group and 9 months in the late intervention group. Enrollment occurred from January to August 2018, and the trial from April 2018 to June 2019.

Consistent with the trial, the study population for this cost-utility study was community-dwelling individuals aged 60 years or older with complex chronic conditions, defined as diagnosed with 2 or more chronic conditions and 10 or more visits to their primary health care provider within the past year. This number of visits has been identified as an indicator of complexity [10]. Chronic conditions were identified through the FHTs electronic medical records. Additional eligibility criteria included the perceived willingness to engage in goal-oriented care conversations, ability to use a smartphone or tablet, capable of providing consent to participate, and willing to complete surveys until completion of the trial. Detailed information on the trial can be found elsewhere [10].

### Ethics Approval

Research ethics approval was granted by the University of Toronto’s Health Sciences Research Ethics Board (approval number 33944) and the ethics committees of all participating practices.

### Intervention and Comparator

The development and usability of the ePRO tool were grounded in user-centered co-design, with a 4-phased approach [13-16]. The ePRO tool has 2 key features: (1) My Goals, which allows patients, caregivers, and providers to create goal-oriented patient care plans using a mobile device during a 15-30-minute care planning appointment. Specified-measurable-attainable-realistic-​time–specific goal principles were used to guide goal setup and include free-form text to write down general feelings on progress; and (2) Outcome Measures, which helps patients, caregivers, and providers to monitor patient measures and outcomes (daily, weekly, or monthly) through validated and reliable health status scales such as Patient-Reported Outcomes Measurement Information System (PROMIS), Global Health Scale (GHS), Health Assessment Questionnaire (HAQ), 9-item Patient Health Questionnaire (PHQ-9) and Generalized Anxiety Disorder 7-Item (GAD-7) scale [9,17-19].

Given that eHealth tools to support self-management are an emerging class of technology, there are no comparable interventions identified for the analysis. Therefore, the standard care comparator in this analysis is multidisciplinary primary care provided by FHTs.

### Time Horizon

The cost-utility analysis compared the costs and outcomes over 15 months of follow-up, which is consistent with the length of the stepped-wedge follow-up period. Costs and health outcomes were not discounted.

### Measurement of Effectiveness

The ePRO effectiveness is measured as the effect of the intervention on HRQoL compared with usual care after adjusting for the family practice sites and multiple measurements at baseline, 3, 6, 9, 12, and 15 months. The trial measured QoL using the Assessment of Quality of Life 4-Dimension (AQoL-4D), a generic instrument that uses multiattribute utility theory, which makes it suitable for cost-utility evaluations [20]. It has also demonstrated validity in chronically ill community-dwelling populations [20]. At each time point, the individual responses of the AQoL-4D questionnaire were converted to weighted multiattributable utility values (ranging from 1.00 [full health] to 0.00 [death equivalent health states]). We used an area under the curve method to estimate quality-adjusted life years (QALYs) and a mixed effect linear regression to estimate the effect of ePRO on QALYs compared with usual care, while controlling for baseline utility values [21], the age of participants, sex, and number of comorbidities and baseline utilities. The baseline QALYs were informed by the observed data during the initial period of the stepped-wedge trial, when all patients received multidisciplinary primary care by Ontario FHTs for 3 or 6 months.

### Resources and Costs

#### Overview

We estimated costs from the perspective of a publicly funded health care system in the province of Ontario, Canada, and considered costs associated with ePRO and health care utilization.

#### Cost of the ePRO Tool

The cost of ePRO tool consisted of technology costs and training costs. Technology-related costs of the intervention were based on real-world costs incurred during the clinical trial. We excluded costs related to trial co-ordination and included any recurrent program costs borne to the government in future adoption. Cost sheets provided by the technology and research partners were stratified by different activities, the quantity used, and the price of each unit. The technology program costs comprised technology support, technology training, licensing, communication, onboarding management, app modification, new feature development, and professional services support costs (Table 1).

**Table 1:** Input parameters of the model.

Parameter	Base estimate (2020 CAD $^a^)	Probability distribution (SD)	Data source
Professional services support	10,284	N/A^b^	ePRO^c^ clinical trial
Technical support (before onboarding)	2971	N/A^b^	ePRO clinical trial
Technical support (after onboarding)	2971	N/A^b^	ePRO clinical trial
License	9182	N/A^b^	ePRO clinical trial
Communication with health care teams	5942	N/A^b^	ePRO clinical trial
Management (before onboarding)	8912	N/A^b^	ePRO clinical trial
Management (after onboarding)	3714	N/A^b^	ePRO clinical trial
Modifications	12,855	N/A^b^	ePRO clinical trial
New features	15,405	N/A^b^	ePRO clinical trial
Total technology costs	72,234	Gamma (11,131)	ePRO clinical trial
Health services utilization per person/year	5500	Gamma (541)	[22]
Total training costs	7233	Gamma (852)	Ministry of Health and Long-Term Care Report [23,24]
Reduction of health services utilization in the standard care group	0.03	Normal (0.003)	[25]
Reduction of health services utilization in the ePRO group	0.04	Normal (0.004)	[25]
Number of patients in the trial	45	N/A	ePRO clinical trial

^a^CAD $1=US $0.80.

^b^N/A: not applicable (used based estimates only).

^c^ePRO: electronic patient reported outcome.

The training costs were included in the analysis as they were considered an opportunity cost. Although they did not represent an additional cost to the public payer, for the time of the training, the clinicians were not in their usual care routine. The training was offered to 5 FHT professionals involved in the delivery of the intervention: family physicians, registered nurses, nurse practitioners, registered social workers, and diabetes educators [26]. The training was administered in 2-hour sessions and was calculated using the hourly wages of the health care providers obtained from published provincial resources. Training costs, together with their sources, for the ePRO intervention are provided in Multimedia Appendix 1.

#### Cost Associated With Health Care Utilization

We obtained the cost associated with health care utilization among patients with complex needs from a retrospective population-based study reporting the health system costs associated with multimorbidity in Ontario [3]. Based on this study, we calculated the total health system costs for older adults living in community with 2-5 comorbidities; these costs were inflated to 2020 values using the health care–specific Consumer Price Index reported by Statistics Canada (Multimedia Appendix 2).

We derived the impact of ePRO on health care system costs based on the 13-item Patient Activation Measure (PAM), a validated tool that provides a weighted score on a scale of 0-100, with 4 associated activation levels, and is used to compare self-management capabilities [27]. In level 1, patients are aware of the importance of their role; in level 2, they have the confidence and knowledge necessary to take action regarding their treatment; in level 3, they actually take action and gain independence; and in level 4, they are able to maintain the behavior even under stress [27]. A score of less than 47.0 places a patient in level 1, 47.1-55.1 level 2, 55.2-72.4 level 3, and more than 72.5 in level 4. Self-management capacity levels, measured using PAM, are associated with reduced utilization across primary and secondary care [25,28,29]. In the absence of administrative and reliable self-reported utilization data, PAM level changes between pre- and postintervention were used to quantify changes in resource use cost from the baseline [25]. Research has demonstrated an inverse correlation between increasing PAM scores and reduction in cost, even after controlling for confounding demographic and comorbidity factors; therefore, it can be used as a credible proxy for future cost savings [25]. We based the percentage of reduction in health services utilization on literature findings of an 8% cost reduction per 1 increase in PAM level change [25]. The PAM questionnaire was administered to patient participants of the study at baseline, 3, 6, 9, 12, and 15 months. To calculate the cost reduction in each group, we multiplied the 8% reduction per level by the percentage of participants in each group that had their PAM levels increased while in that state. For example, if in one of the groups 10% of patients achieved a 1-level increase and 20% achieved a 2-level increase over the course of the trial, the total health system costs for that group were reduced by 24% (0.08 × 0.1 + 0.16 × 0.2).

### Analysis

We used a decision tree to estimate the expected total costs and outcomes associated with ePRO and usual care after consultation with clinical partners. The tree splits into 2 branches, ePRO tool intervention and usual care. The incremental cost-effectiveness ratio of ePRO versus usual care was calculated by dividing the differences in total costs by the difference in QALYs. We conducted sensitivity analyses to address the uncertainties of our model and to better understand the impact of model assumptions on cost-effectiveness results, including a tornado diagram for the incremental net monetary benefit at a CAD $50,000 (~US $40,000)/QALY willingness-to-pay (WTP) threshold, and a probabilistic sensitivity analysis with 10,000 Monte Carlo simulations, sampled using the related distribution of the parameters (Table 1).

## Results

### Base Case Analysis

The total cost of the ePRO tool was CAD $79,467 (~US $63,581; CAD $1733 [~US $1386] per person); of these, the technology component accounted for 90.89% (CAD $72,234 [~US $57,794]) of the total costs. The trial reported that ePRO combined with usual multidisciplinary care did not significantly impact patient HRQoL (*P*=.24) or patient activation (*P*=.17) [10]. Our base case analysis showed that, compared with the standard care, the addition of the ePRO intervention was associated with higher costs (CAD $7133 [US $5707] vs. CAD $5423 [US $4338] per patient) and slightly fewer QALYs (0.42 vs. 0.45) than usual care (Table 2). The technology cost was a key driver of an incremental cost. Although the ePRO intervention could reduce health system costs compared with standard care (CAD $5258 [US $4206] vs. CAD $5324 [US $4259], respectively), this saving was insufficient to offset the added technology cost.

**Table 2:** Base case results.

Strategy	Mean costs (CAD $^a^)	Incremental costs (CAD $)	Mean QALYs^b^	Incremental QALYs	ICER^c^ (CAD $/QALY)
Usual care	5423	—	0.45	—	—
ePRO^d^	7133	1710	0.42	–0.03	Dominated^e^

^a^CAD $1=US $0.80.

^b^QALY: quality-adjusted life year.

^c^ICER: incremental cost-effectiveness ratio.

^d^ePRO: electronic patient-reported outcome.

^e^ePRO was more costly and produced fewer QALYs than usual care.

### Sensitivity Analyses

**Figure 1:**
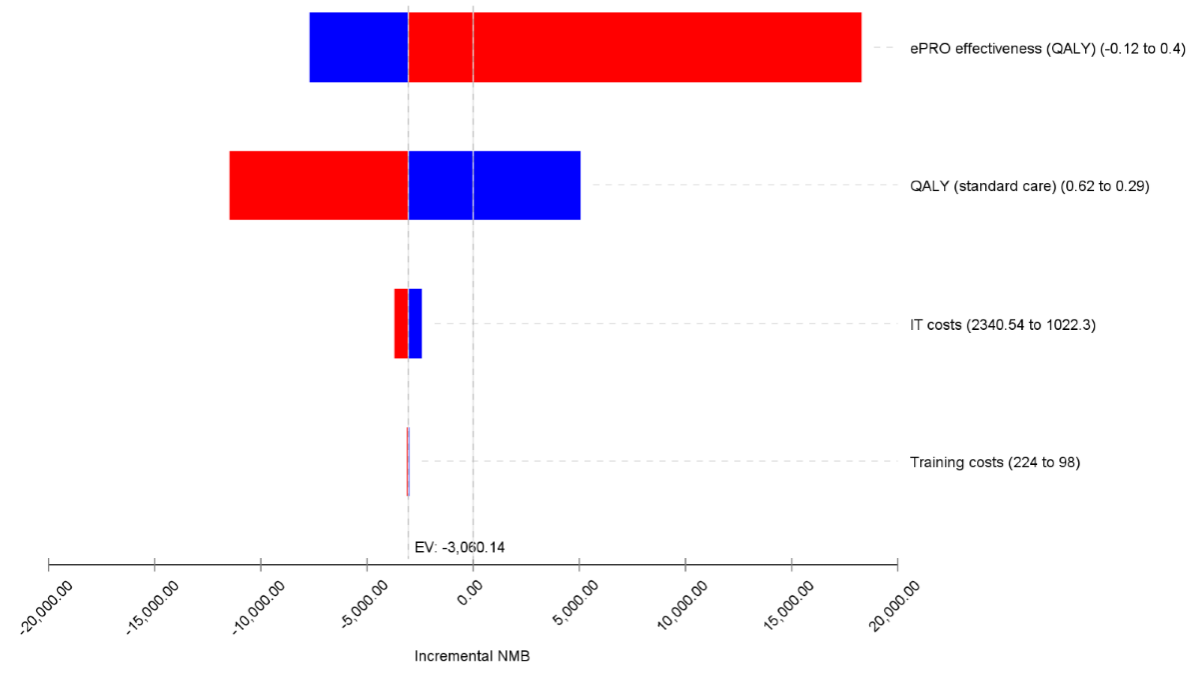
One-way sensitivity analysis (tornado diagram). ePRO: electronic patient-reported outcome; EV: expected value; IT: information technology; NMB: net monetary benefit; QALY: quality-adjusted life year;.

**Figure 2:**
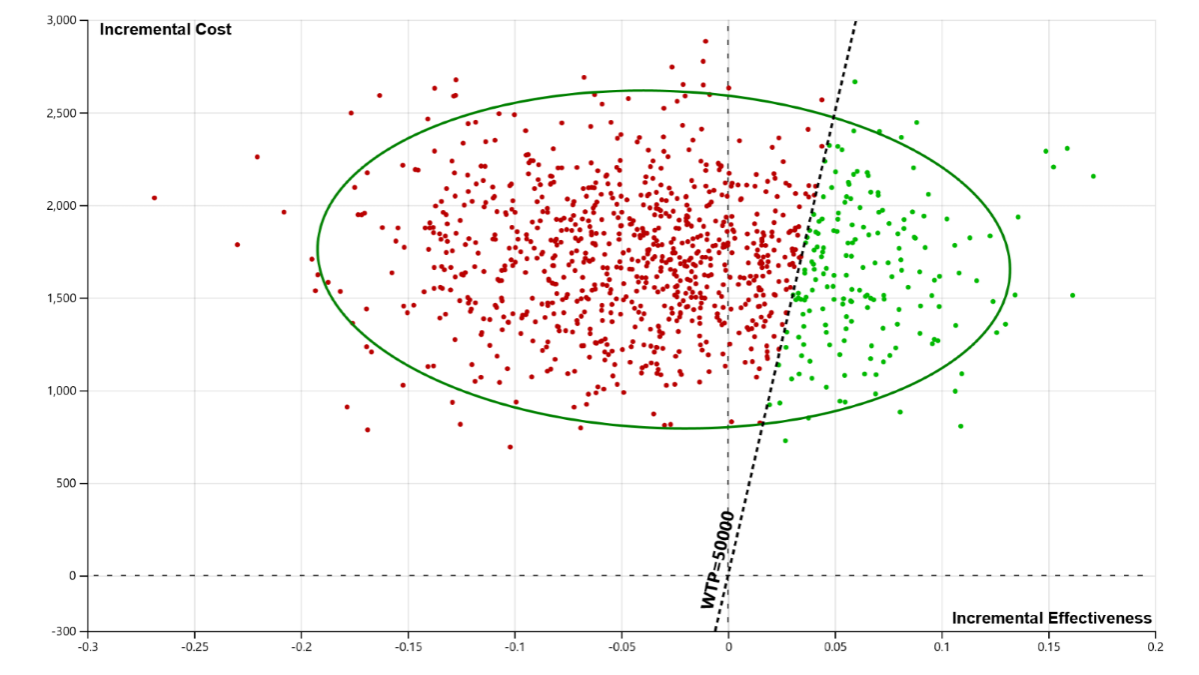
Probabilistic sensitivity analysis. WTP: willingness-to-pay threshold.

**Figure 3:**
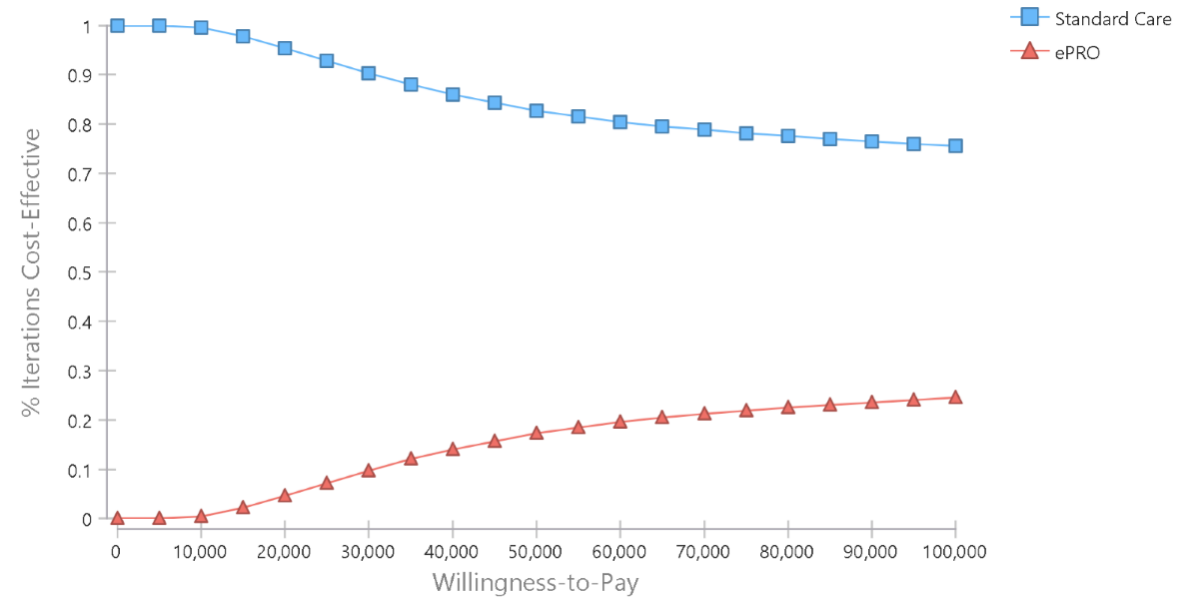
Cost-effectiveness acceptability curve. ePRO: electronic patient-reported outcome.

Results of the deterministic sensitivity analysis showed that the effectiveness of ePRO is the most influential driver of the cost-effectiveness findings. ePRO would be considered a cost-effective option, that is, having a positive incremental net monetary benefit, if it could improve by at least 0.03 QALYs. Other key determinants included the technology and the training costs for the implementation of the tool. However, individual variation in none of these costs could change the cost-effectiveness results (Figure 1). The probabilistic sensitivity analysis results showed that ePRO has a 17.3% probability of being cost-effective at the WTP threshold of CAD $50,000 (~US $40,000)/QALY (Figures 2 and 3); this probability increased to 25.1% if the WTP threshold increased to CAD $100,000 (~US $80,000)/QALY.

## Discussion

### Principal Findings

Our study showed the ePRO tool was not cost-effective at a commonly used WTP threshold of CAD $50,000 (~US $40,000)/QALY. The main driver of our cost-effectiveness results is the effectiveness of the ePRO tool on QALYs. Our results were robust to changes in input parameters and model assumptions with the probability of being cost-effective of 17.3% at the WTP values of CAD $50,000 (~US $40,000)/QALY.

Previous studies assessing the cost-effectiveness of eHealth interventions reported mixed findings, where some interventions were considered cost-effective or cost-saving, whereas other studies reported that eHealth interventions were not cost-effective [11,30]. Some economic evaluations of these technologies also reported comparable HRQoL among patients receiving eHealth and those receiving usual care [31-33]. Consistent with these results, our study showed that the addition of ePRO to usual care was not cost-effective because the tool generated fewer QALYs than usual care. Lower QALYs observed in this cost-utility analysis mirrored insignificant changes in HRQoL that might be due to the lack of statistical power resulting from recruitment challenges and low response rates to the surveys [31-33].

Some aspects of the ePRO tool must be considered when interpreting this economic evaluation. The ePRO tool is not targeted to patients with a specific disease, but rather to a heterogeneous population with a different number and variety of conditions. Hence, effectiveness of the ePRO tool may be different for patients with different conditions, given their complex needs and individual preferences [11]. Accessibility and digital literacy also have a direct impact on the effectiveness of eHealth interventions, as they can increase access and be convenient for some patients, but may also be inappropriate or inaccessible to others [7].

Furthermore, being a tool that is mainly focused on improving the patient’s engagement in the treatment and changing health behaviors, a trial-based cost-effectiveness analysis may not be the ideal design as it may take more than 10 years for health interventions to change patients’ behaviors and respective health outcomes [9,11,34]. With prolonged use of the tool, more significant effects may be detected, especially in chronic conditions that are mostly affected by changes in the behavior of the patients. Additional outcomes, such as patient and provider satisfaction, disease management, and self-care activities, with a longer time horizon could be useful in identifying whether ePRO had any other relevant benefits. Previous economic evaluations of eHealth technologies with no impact in clinical outcomes concluded that they were cost-effective due to their low cost and effectiveness when considering outcomes beyond HRQoL [32,35]. eHealth interventions can also have gains in efficiency with the increase in the number of users and sharing of data systems and infrastructure [31].

Another relevant consideration is that with prolonged use of the app and in less controlled conditions, patient adherence may decrease [36]. Data collected during the stepped-wedge trial indicated that adherence to the tool was moderate; however, this does not necessarily translate into fidelity to the model of care—a shift from the more classic, passive behavior of the patient toward their health to a model with higher engagement of both providers and patients in goal-setting conversations and oriented care [37,38]. It is possible that even with patients continually using the tool, the expected effects on health outcomes may not fully materialize if this shift in the model of care does not occur.

### Study Limitations

This analysis had some important limitations. First, our economic evaluation was based on a single stepped-wedge trial, which limits the generalizability of our study for the real-world setting and a more heterogeneous population. Despite being a pragmatic trial that intended to approximate as much as possible the usual care conditions, the trial faced recruitment challenges and low response rates to outcome measures. Moreover, the trial was conducted within the context of FHTs, but ePRO could also be applicable to other models of primary care. The use of health care payer perspective in this analysis excludes some benefits that can add value to the ePRO tool. A societal perspective would allow the inclusion of indirect costs such as informal and unpaid time in caregiving, increased poverty, and loss of income due to time away from work [6]. For an intervention focused on primary care, out-of-pockets costs may be relevant, especially in health systems similar to Canada, where hospital-level costs are publicly funded, but many outpatient and nonphysician services are not funded. Some eHealth interventions were found to be cost-effective due to the inclusion of costs pertinent to a societal perspective [39,40].

Lastly, we used the PAM scores as a proxy to calculate the reduction in health care service utilization costs of the patients in the trial. Although an increase in patient activation levels has been associated with a reduction in these costs, these values can vary, and this was not tested in the Canadian setting [25,29]. However, our sensitivity analysis showed that our results were robust to a change in the effect of the ePRO tool on health care utilization.

### Conclusion

Our cost-utility analysis highlighted that the ePRO tool is not a cost-effective technological solution for community-dwelling older adults with multiple chronic conditions when compared with usual care. However, the tool would become a cost-effectiveness option if it could improve QALYs by at least 0.03 unit. This study highlights the minimal effectiveness of eHealth solutions required to make the solutions cost-effective in response to the rising trends of complex, aging populations within finite-resourced health systems. Fidelity and adherence to the eHealth tools could improve their effectiveness, and this relationship should be investigated in future studies. Pragmatic trials with larger number of participants, fewer missing data, and longer follow-up time could help inform the implementation of the eHealth intervention, such as ePRO tool, in a finite-resourced setting.

## Appendix

Multimedia Appendix 1 Family health team provider training costs.

Multimedia Appendix 2 Health care utilization costs (person-level costing using administrative databases in Ontario).

## Abbreviations

AQoL-4D: The Assessment of Quality of Life 4-Dimension
ePRO: electronic patient-reported outcome
FHT: family health team
GAD-7: Generalized Anxiety Disorder 7-Item scale
GHS: Global Health Scale
HAQ: Health Assessment Questionnaire
HRQoL: health-related quality of life
ICER: incremental cost-effectiveness ratio
PAM: Patient Activation Measure
PHQ-9: 9-item Patient Health Questionnaire
PROMIS: Patient-Reported Outcomes Measurement Information System
QALY: quality-adjusted life year
WTP: willingness to pay
