# Supplementary material for: An Electronic Patient-Reported Outcomes Tool for Older Adults With Complex Chronic Conditions: Cost-Utility Analysis
Source: JMIR Aging. 2022 Apr 20;5(2):e35075. doi: 10.2196/35075 (PMC9069297; doi:10.2196/35075)
Supplement: Multimedia Appendix 1 [file aging_v5i2e35075_app1.docx]

##

| Provider Type | Ministry Unit Cost Description | Hourly Wage (CAD $) | Source |
| --- | --- | --- | --- |
| Family Medicine Physicians | Patient Enrollment Model - 3.4 Days per Week | 203.51 | *OLA 2015/2016 Standing Committee on Public Accounts: Physician Billing ^a^* |
|  | Patient Enrollment Model – 40-hour work week (FTE) | 299.60 |  |
| Registered Nurses | Salary - 40-hour work week (FTE) | 34.96 | *OLA Family Health Teams: 2009 Guide to Interdisciplinary Provider Compensation ^a^* |
| Nurse Practitioners | Salary - 40-hour work week (FTE) | 48.00 |  |
| Registered Social Workers | Salary – 40-hour work week (FTE) | 34.96 |  |
| Diabetes Educators | Salary – 40-hour work week (FTE) | 32.68 |  |

*^a^ price inflated to 2020 prices using inflation using the Bank of Canada’s target inflation rate.*

*FTE = Full Time Equivalent (FTE) refers to the unit of measurement equivalent to an individual of one unit of a work, applicable in a variety of context.*
