# Supplementary material for: An Electronic Patient-Reported Outcomes Tool for Older Adults With Complex Chronic Conditions: Cost-Utility Analysis
Source: JMIR Aging. 2022 Apr 20;5(2):e35075. doi: 10.2196/35075 (PMC9069297; doi:10.2196/35075)
Supplement: Multimedia Appendix 2 [file aging_v5i2e35075_app2.docx]

| Type of Service | Utilization Database | Weight Name | Unit Cost |  |
| --- | --- | --- | --- | --- |
| Group 1: Short Episodes (mean <60 Days) | | | |  |
| Acute Hospitalization | DAD: Discharge Abstract Database | Resource Intensity Weight (RIW) | Cost per Weighted Case (CPWC) | |
| Same Day Surgery & Outpatient | NACRS: National Ambulatory Care Reporting System | Resource Intensity Weight (RIW) | Cost per Weighted Case (CPWC) | |
| Emergency Department | NACRS: National Ambulatory Care Reporting System | Resource Intensity Weight (RIW) | Cost per Weighted Case (CPWC) | |
| Inpatient Rehabilitation | NRS: National Rehabilitation Reporting System | Rehabilitation Cost Weight (RCW) | Cost per Weighted Case (CPWC) | |
| Group 2: Longer Term Episodes | | | |  |
| Complex Continuing Care | CCRS: Continuing Care Reporting System | Case Mix Index | Cost per RUG (Resource Utilization Groups) -Weighted Patient Day: CPRWPD | |
| Inpatient Mental Health | OMHRS: Ontario Mental Health Reporting System | SCIPP CMI: System for Classification of In-Patient Psychiatry Case Mix Index | Cost per RUG (Resource Utilization Groups) -Weighted Patient Day: CPRWPD | |
| Group 3: Visits/Claims | | | |  |
| Primary Care Physician | OHIP | n/a | Fee Paid | |
| Specialist Care Physician | OHIP | n/a | Fee Paid | |
| Home Care | OHCAS, HCD: Ontario Home Care Administrative System and Home Care Database | n/a | Cost per Visit | |
| Pharmaceuticals | ODB: Ontario Drug Benefit | n/a | Amount Paid | |
| Equipment | ADP: Assistive Devices Program | n/a | Amount Paid | |

*The aforementioned study followed the guidelines on person-level costing implemented at ICES, which was developed by a co-authored of population study (WW) and also is a principal investigator of the ePRO project*
